# Investigation of the Ternary System, Water/Hydrochloric Acid/Polyamide 66, for the Production of Polymeric Membranes by Phase Inversion

**DOI:** 10.3390/membranes15010007

**Published:** 2025-01-01

**Authors:** Jocelei Duarte, Camila Suliani Raota, Camila Baldasso, Venina dos Santos, Mara Zeni

**Affiliations:** 1 Postgraduate Program in Process and Technologies Engineering (PGEPROTEC), University of Caxias do Sul, Caxias do Sul 95070-560, RS, Brazil; cbaldasso@ucs.br (C.B.); vsantos2@ucs.br (V.d.S.);; 2 Postgraduate Program in Materials Science and Engineering (PPGMAT), University of Caxias do Sul, Caxias do Sul 95070-560, RS, Brazil; csraota@ucs.br

**Keywords:** polymeric solution, interaction parameter, phase diagram, phase inversion

## Abstract

The starting point for the preparation of polymeric membranes by phase inversion is having a thermodynamically stable solution. Ternary diagrams for the polymer, solvent, and non-solvent can predict this stability by identifying the phase separation and describing the thermodynamic behavior of the membrane formation process. Given the lack of data for the ternary system water (H_2_O)/hydrochloric acid (HCℓ)/polyamide 66 (PA66), this work employed the Flory–Huggins theory for the construction of the ternary diagrams (H_2_O/HCℓ/PA66 and H_2_O/formic acid (FA)/PA66) by comparing the experimental data with theoretical predictions. Pure polymer and the membranes produced by phase inversion were characterized to provide the information required to create the ternary diagrams. PA66/FA and PA66/HCℓ solutions were also evaluated regarding their classification as true solutions, and the universal quasi-chemical functional group activity coefficient (UNIFAC) method was used for determining non-solvent/solvent interaction parameters (*g*_12_). Swelling measurements determined the polymer/non-solvent interaction parameter (*χ*_13_) for H_2_O/PA66 and the solvent/polymer interaction parameter (*χ*_23_) for PA66/FA and PA66/HCℓ. The theoretical cloud point curve was calculated based on “Boom’s LCP Correlation” and compared to the curve of the experimental cloud point. The ternary Gibbs free energy of mixing and *χ*_23_ indicated FA as the best solvent for the PA66. However, for HCℓ, the lower concentration (37–38%), volatility, and fraction volume of dissolved PA66 (*ϕ*_3_) indicated that HCℓ is also adequate for PA66 solubilization based on the similar membrane morphology observed when compared to the PA66/FA membrane.

## 1. Introduction

Polyamide 66 (PA66) is one of the most valuable engineering thermoplastics due to its good thermal stability and high mechanical and chemical resistance [1]. It is an aliphatic and semi-crystalline polymer obtained by the polycondensation of adipic acid and 1,6-hexamethylene diamine. PA66 has many applications, including the preparation of polymeric membranes applied in membrane separation processes (MSPs) [2,3,4].

The phase inversion method is one of the most often used to produce polymeric membranes, in which the solvent is usually removed from the polymeric solution through contact with a non-solvent [5,6,7,8]. When a polymeric solution meets a non-solvent, the solvent exchange by the non-solvent modifies the polymeric solution composition, inducing phase separation. Thus, two equilibrium phases are formed: polymer-rich and polymer-lean [9]. This phase separation is named liquid–liquid (L–L) demixing. The (L–L) demixing competes with the solid–liquid demixing (S–L) [10,11], which occurs when the polymer precipitates from the initial polymeric solution [9].

The initial polymeric solution, when constituted by liquid systems with polymers of low molecular weight, may be classified as a true solution when it has the following: (i) affinity between the components, (ii) spontaneous formation, (iii) constant concentration over time, (iv) homogeneity, and (v) thermodynamic stability [12]. The typical polymer concentration in true solutions for membrane preparation is of the semi-diluted classification (concentration between 10 and 30%) but can also be diluted (≤2%) or concentrated (≥50%) [13,14].

**Figure 1:**
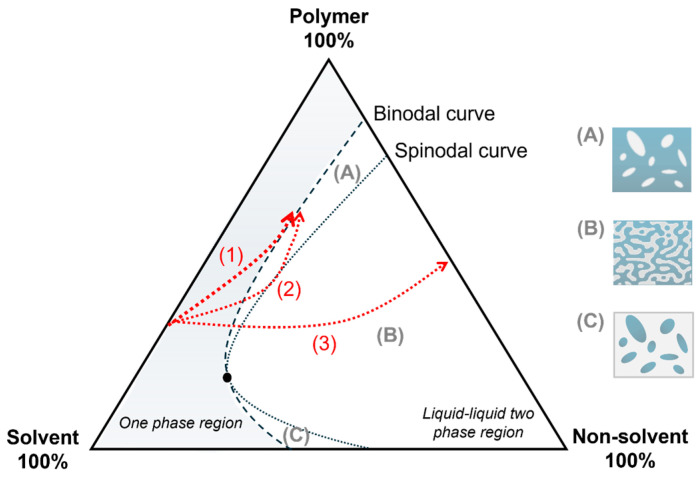
Typical schematic phase diagram of a ternary system: (1) composition path of delayed L–L demixing; (2) composition path of instantaneous L–L demixing; (3) spinodal decomposition; (**A**) nucleation of the polymer-lean phase (binodal demixing); (**B**) bicontinuous structure (spinodal demixing); and (**C**) nucleation of the polymer-rich phase (binodal demixing).

A typical representation of a ternary phase diagram is shown in Figure 1 [15,16,17], which generally includes the binodal and spinodal lines. The binodal line delimits the homogeneous phase region (one-phase region), while the spinodal line demarcates the liquid–liquid (two-phase) [6]. The region between the binodal and spinodal lines is the metastable region, and the critical point is the intersection of the binodal and spinodal lines [18].

The analysis of the ternary diagram can predict the phase separation and, consequently, the final morphology of the membrane [18]. The precipitation is instantaneous in the L–L demixing (Line 2, Figure 1), and the phase separation occurs immediately. The fast phase separation forms an interfacial layer with higher polymeric concentration, which acts as an additional resistance to mass transfer between the bath and the sublayers of the polymeric solution. This process favors the formation of pores and their interconnectivity [19], as well as a thin top layer (skin), which is dense or with some degree of porosity [17].

It is also possible to have a delayed onset L–L demixing (Line 1, Figure 1), which requires a contact time between the solution and the non-solvent to promote the phase separation. In delayed onset precipitation, the membranes usually have a porous substructure of closed cells with a thin and dense skin, promoting flow resistance. The degree of porosity and interconnectivity of the pores in this kind of membrane are generally low [5].

The composition path can be changed with extended precipitation time, as observed in Line 3 of Figure 1 [20]. Furthermore, according to the position where the L–L demixing in the ternary phase diagram occurs, three composition path possibilities can happen when crossing the binodal line: (i) under the critical point, (ii) above the critical point, or (iii) through the critical point. The first happens with solutions of low polymer concentration, in which the demixing by nucleation of the polymer-rich phase creates droplets of concentrated polymer, resulting in a latex-like structure (Figure 1C) [3]. In the second possibility, the demixing occurs by nucleation of the polymer-lean phase, forming a porous structure due to the droplets of lower polymer concentration (Figure 1A). If the droplets bond before the polymer-rich phase solidifies, an open porous system will be formed. The third possibility causes the instantaneous L–L separation because it directly reaches a metastable region, forming a membrane with a bicontinuous morphology of interlaced droplets of lower and higher polymer concentrations (Figure 1B) [6].

Given its relevance in membrane preparation, the study of ternary systems of polymers with solvent and non-solvent polyamide has been gaining relevance in the literature [9,18,21,22]. However, ternary systems of polyamide 66 are still scarce. Bulte et al. (1993), for example, reported the S–L demixing mechanism as favorable when solutions with concentrations greater than 17% of 4,6 aliphatic polyamide in formic acid (FA), while the L–L demixing mechanism took place at lower polymer concentrations [11]. However, the S–L mechanism was noted as kinetically competitive with the L–L mechanism when the initial solution contained small nucleators (due to the polymer crystallinity) [11]. Interestingly, the studies carried out by Bulte et al. (1996) described membranes with morphology typical of the L–L demixing processes but obtained from more thermodynamically favorable S–L demixing processes from ternary systems [23,24].

Shih et al. (2005) observed the morphology of membranes precipitated in water from Nylon 6/FA solutions, a system with potential instability of L–L demixing and crystallization [25]. These processes can occur sequentially or simultaneously, depending on the forces acting on the system. The dissolution of polyamide in a good solution (i.e., solutions that protonate polyamides, such as FA solutions) causes slow crystal nucleation and an initial L–L demixing mechanism. On the other hand, a mild precipitation bath (not as aggressive as water) promotes the crystallization part of S–L process, resulting in a thin skin microporous membrane. Similar behavior was observed by Lin et al. (2006) in determining the degree of crystallinity in Nylon 66 membranes prepared by phase inversion in water/FA [3].

Thomas et al. (2002) analyzed the compositional crystallization trajectory of polyamide 6 (PA6) membranes solubilized in FA [26]. They observed the precipitation beginning at a certain distance from the interface between the membrane and the non-solvent bath and then propagating up and downwards [26]. According to the authors, the membrane formation process is very complex, and the crystallization rate may depend on kinematic (e.g., viscosity) and thermodynamic (e.g., composition and distance from crystallization boundary phase) factors. In addition, working with PA6 and FA, El-Gendi et al. (2012) constructed the ternary phase diagram PA6/FA/H_2_O through experimental turbidity measurements during titration of PA6/FA solution in water [27]. The authors highlighted that a large amount of water (26–39 wt%) was needed to achieve precipitation and L–L phase separation [27].

The membrane morphology is also dependent on the choice of the solvent/non-solvent system, which interferes with the phase separation [9,17,21]. A higher affinity between solvent and non-solvent promotes porous membranes, while dense membranes and non-porous top layers are obtained in cases of lower affinity [6]. The thermodynamic properties of polymeric solutions also affect the membrane’s formation and morphology [6,8,28,29]. The membrane formation is also affected by the mass transfer between the coagulation bath and polymeric solution caused by the difference in the chemical potential (driving force) of the components [6]. Thus, the comprehension of parameters that influence the phase inversion leads to a better understanding of the processes [6,22,28,29].

A practical way to understand and predict the behavior and properties of polymeric solutions is to use models, which are usually classified into two categories: the lattice model and the van der Waals model. These two approaches can be used to derive the activity coefficient models or state equations [30]. The Flory–Huggins dimensionless parameter “*χ*” has been widely used to characterize solvent–polymer and polymer–polymer interactions. While the original theory proposed that *χ* is concentration-independent, many solvent–polymer systems exhibit increased *χ* with increasing polymer concentration [31]. More than that, the advantages of using the Flory–Huggins model are its simplicity, wide acceptance and familiarity, and the requirement of only the molar volumes of components [18]. The other variables are eliminated by using empirical parameters, and even though this practice diminishes the theoretical basis of the model, the results justify this practical approach [32].

Bulte et al. (1996) investigated the ternary system nylon/FA/H_2_O by using the Flory–Huggins model, determining the constants for Nylon 4,6, Nylon 4,6-co-6, and Nylon 6 [23]. A similar study was performed by Cheng et al. (1994) for Nylon 6, Nylon 66, and Nylon 610 with FA and H_2_O [33]. Other publications have explored ternary systems for polyamide/FA/H_2_O but have not focused on determining the interaction parameters. These investigations indicated the precipitation way [3]; calculated the concentration profile, precipitation times, and diffusion trajectories [34]; identified the three relevant regions, the binodal boundaries, and the crystallization line [25]; investigated the morphology as a function of precipitation conditions [27]; and characterized the membranes obtained and indicated the crystallization limits [35].

Given the lack of data on HCℓ/PA66 due to the HCℓ classification as a solution instead of a pure solvent, this work aimed to create the ternary system H_2_O/HCℓ/PA66. For this, the parameters for HCℓ and HCℓ/PA66 were determined, as well as the study evaluation of the system as a true solution. The data were then employed in the Flory–Huggins model and the cloud point curve to build the ternary system H_2_O/HCℓ/PA66. The characterization results and the ternary system were compared to the existent H_2_O/FA/PA66 system, the most used system for PA66 membrane preparation via the phase inversion method. In addition, the thermodynamic properties of both systems (H_2_O/HCℓ/PA66 and H_2_O/FA/PA66) were correlated to the resultant PA66 membrane morphology, which is a relevant parameter since it interferes with the membrane performance.

## 2. Materials and Methods

### 2.1. Materials

The polymer used was the commercial polyamide 66 (PA66, pellets) supplied by Alfa Chemical, Co., Ltd. (Zhengzhou, China). PA66 was dried for 2 h at 90 °C before each procedure to remove any adsorbed water that could influence the analysis [36]. The solvents used in the preparation of solutions and membranes were fuming hydrochloric acid (HCℓ, 37–38%) and formic acid (FA, 98–100% and 90%). The 90% purity FA was used in the viscosimetric molar mass determination of PA66, according to ISO 307:2007(E) [37]. All solvents were supplied by Merck (Rio de Janeiro, Brazil). Ultrapure water was produced from a Milli-Q system. Further information about the properties of the polymer and solvents is listed in Table 1.

Unless otherwise stated, the procedures were performed at an environment-controlled temperature of 25 ± 2 °C and under atmospheric pressure (1 atm).

### 2.2. Polymer Characterization: Water Adsorption

The water content absorbed by PA66 was determined according to the ASTM D570-98 (2010) ^ε1^ adapted method [39]. PA66 films (100 mm × 100 mm × 1 mm) were pressed (6 t, 5 min, 270 ± 2 °C) and dried at 110 °C for 24 h. The membranes were weighed (*m_i_*, g) and placed in a beaker containing distilled water for 24 h. The excess water was removed from the membranes with a paper towel, and the samples were weighted again (*m_f_*, g). The water content was determined using Equation (1), correlating it with the humidity (%).

(1) $$Humidity=\frac{\left(m_{f}-m_{i}\right)}{m_{i}}\times 100$$

### 2.3. Membrane Preparation

The membranes were obtained by the immersion precipitation technique [6] of the two systems PA66/FA/H_2_O and PA66/HCℓ/H_2_O. A schematic of the membrane preparation is presented in Figure S1 (Supplementary Information). First, the PA66/HCℓ and PA66/FA solutions were prepared by dissolving 20 g of PA66 in 100 cm^3^ of each solvent under magnetic stirring for 2 h (Figure S1, Step 1). The stirrer was insulated with glass wool to avoid heat transfer by friction, guaranteeing the solution concentration due to the volatility of the solvent.

The solution was spread on a glass support using a casting knife to form a film with a wet thickness of ~0.3 μm (Figure S1, Step 2). Subsequently, the solvent was evaporated at a controlled temperature (depending on the solvent used) (Figure S1, Step 3) and then immersed in the precipitation bath (Table 2) (Figure S1, Step 4).

### 2.4. Membrane Characterization

The membranes’ performances were evaluated in previous publications by means of permeability and selectivity [40,41]. In brief, the membrane produced with FA showed a pure water flux of 39 L m^−2^ h^−1^, while a pure water flux of 5.5 L m^−2^ h^−1^ was obtained for the one prepared with HCℓ, when both membranes were compacted for 2 h at 101 kPa [41]. When compacted at 1500 kPa, the pure water flux was 17 and 22.2 L m^−2^ h^−1^ for FA and HCℓ, respectively [40]. The selectivity was evaluated regarding egg albumin (45 kDa) and bovine serum albumin (69 kDa), and both membranes presented similar selectivity values of 70 and 80%, respectively.

#### 2.4.1. Fourier-Transform Infrared Spectroscopy Analysis

PA66 membranes and pure PA66 were analyzed by Fourier-transform infrared spectroscopy (FTIR) to evaluate the effect of the solvents in the PA66 structure. The membranes were analyzed using the Attenuated Total Reflectance (ATR) accessory, while pure PA66 was analyzed in KBr tablets. The analysis was performed in a Nicolet IS10-Thermo Scientific (Waltham, MA, USA) using the transmittance mode, with a range of 4000 to 400 cm^−1^ and a 4 cm^−1^ resolution.

#### 2.4.2. Differential Scanning Calorimetry Analysis

The thermal analysis through differential scanning calorimetry (DSC) was performed using Shimadzu DSC-50 equipment (Kyoto, Japan). PA66 (pellets) was cryogenically milled and dried under vacuum at 90 °C for 2 h, while PA66/HCℓ membranes were analyzed in film shape. The analyzed sample mass was 10 mg, with a heating and cooling rate of 10 °C·min^−1^ under a nitrogen atmosphere (flux of 50 mL·min^−1^) [42]. The melting enthalpy values (Δ*H_f_*, J·g^−1^) and the melt temperature (*T_f_*, °C) were taken from the curves generated in the second heating. The crystallinity degree (*X_c_*, %) was calculated from Equation (2), adopting the value of 196 J·g^−1^ as the theoretical melting enthalpy of 100% crystalline PA66 (Δ*H*^0^*_f_*) [36].

(2) $$X_{c}=100\times \left(\frac{{∆H}_{f}}{{∆H}_{f}^{0}}\right)$$

#### 2.4.3. Scanning Electron Microscopy

The scanning electron microscopy (SEM) analyses of surface and cross-section membranes were performed using a Shimadzu microscope, model SSX 550 (Kyoto, Japan). The samples were previously fractured in liquid nitrogen and metallized with a thin layer of gold by means of sputtering. The analysis employed the secondary electrons’ detector, an acceleration voltage of 15 kV, and magnifications of 2000 and 1000 times.

### 2.5. Solution Characterization

The complete study about the evaluation of the PA66/HCℓ system as a true solution involved the determination of the following: (i) affinity between components, (ii) solution spontaneous formation, (iii) constant concentration over time, (iv) homogeneity, and (v) swelling. All the detailed methodologies are described in a previous publication [43]. In brief, the results indicated affinity between PA66 and HCℓ, with slow diffusion among the polymer chains conferring mobility and creating a homogeneous solution with no phase separation over time. In addition, HCℓ vapor promoted unlimited PA66 swelling, i.e., dissolving the polymer before it swelled.

### 2.6. Polymer Binary and Ternary Systems Determination

For the construction of a ternary phase diagram, the determination of three binary interaction parameters is necessary: the non-solvent/solvent system (*g*_12_), the non-solvent/polymer (*χ*_13_), and the solvent/polymer (*χ*_23_). The subscripts “1”, “2”, and “3” refer to the non-solvent, solvent, and polymer, respectively.

#### 2.6.1. Determination of the Non-Solvent/Solvent Interaction Parameter (*g*_12_)

The *g*_12_ binary parameter interaction for a non-ideal system is usually determined from the excess Gibbs free energy (*G^E^*) by Equation (3) [44].

(3) $$G^{E}={∆G}_{m}-RT(x_{1\;}{lnx}_{1}+x_{2}{lnx}_{2})$$

where Δ*G_m_* is the Gibbs free energy of mixing, *R* is the universal gas constant, *T* is the absolute temperature, and *x* is the component’s molar fraction.

For a two-component system, *G^E^* is related to the Gibbs free energy of the mixture by Equation (4) and can generally be obtained by vapor pressure experiments or liquid–vapor equilibrium data [45,46].

(4) $$\frac{{∆G}_{m}}{RT}=x_{1}{ln}_{\varphi 1}+x_{2}{ln}_{\varphi 2}+g_{12}(\varphi )x_{1}\varphi_{2}$$

where *φ* is the volumetric fraction.

The *g*_12_ parameter can be considered a free energy term, containing both enthalpic and entropic contributions. As can be seen in Equation (5), the interaction parameter is concentration-dependent; thus, the symbol *g* replaces *χ*.

(5) $$g_{12}=\frac{1}{x_{1}\varphi_{2}}\left[x_{1}ln\frac{x_{1}}{\varphi_{2}}+x_{2}ln\frac{x_{2}}{\varphi_{2}}+\frac{G^{E}}{RT}\right]$$

The non-solvent/solvent interaction parameter was determined using the cloud point method. The cloud point curve was determined by the titration method [44,47]. For this, PA66 solutions with concentrations of 5, 10, 15, 20, 25 and 30 g in HCℓ and in FA were prepared. Distilled water (non-solvent) was slowly added to the solution with a burette until the solution went from totally translucid to cloudy. After the first turbidity signal, the non-solvent addition was discontinued, and the solution remained under stirring for a further 30 min to verify the turbidity stability. In the case the solution returned to be translucid, the procedure of adding water was resumed. The cloud point curve composition was then determined by the non-solvent/solvent/polymer amount.

The fit tests for the experimental data obtained in the cloud point curve experiments were conducted using the cloud point curve linearization (CPL) method. Using the CPL Boom’s correlation method, the behavior of the calculated cloud point curve was evaluated by Equation (6) in relation to the experimental data. If experimental data are well fitted by the CPL Boom’s method, the L–L demixing process occurs; otherwise, the S–L happens.

(6) $$ln\frac{w_{1}}{w_{3}}=bln\frac{w_{2}}{w_{3}}+a$$

where *w*_1,2,3_ are the component’s mass fraction. The intersection (*a*) obtained through the linearization of Equation (6) is related to interaction parameters and molar volumes, i.e., enthalpic interactions are considered. The linearization slope (*b*) relates only to entropic parameters, i.e., molar volumes.

The liquid–vapor equilibrium data for the H_2_O/FA system are available in the literature; however, they are not found for the H_2_O/HCℓ system. So, from non-solvent/solvent amounts added in the different solutions, the Aspen Plus 7.3 software was used to estimate the liquid–vapor equilibrium values using the Universal Functional Activity Coefficient (UNIFAC) [47,48,49] method for both systems (HCℓ and FA). The software determined the Gibbs free energy of mixing for the systems, and *g*_12_ was then calculated by Equation (5).

In addition, the Flory–Huggins theory was used to describe the phases’ behavior in ternary systems of polymeric solutions for membrane formation, in which binary interaction parameters were included. According to this theory, the Gibbs free energy of the mixture (Δ*G_m_*) for ternary systems is provided by Equation (7).

(7) $$\frac{{∆G}_{m}}{RT}=n_{1}ln\varphi_{1}+n_{2}ln\varphi_{2}+n_{3}ln\varphi_{3}+g_{12}n_{1}\varphi_{2}+\chi_{13}n_{1}\varphi_{3}+\chi_{23}n_{2}\varphi_{3}$$

where *n*_1,2,3_ are the molar number, *φ*_1,2,3_ are the volumetric fraction of the components, *R* is the gas constant, and *T* is the absolute temperature (K).

#### 2.6.2. Determination of the Non-Solvent/Polymer Interaction Parameter (χ_13_)

The non-solvent/polymer interaction parameter was determined by swelling in equilibrium [6,44]. PA66 film samples were prepared with dimensions of 100 mm × 100 mm × 1 mm, pressed (6 t, 5 min, 270 ± 2 °C), and dried in oven at 110 °C for 24 h. The samples were kept submerged in a beaker containing distilled water under a controlled temperature of 25 ± 2 °C. Every two days, the samples were taken out of the water and weighed. This procedure was repeated until no significant difference in mass was observed between measurements. The non-solvent/polymer interaction parameter was then calculated using Equation (8).

(8) $$\chi_{13}=-\frac{ln\left(1-\varphi_{3}\right)+\varphi_{3}}{{\left(\varphi_{3}\right)}^{2}}$$

where *φ*_3_ is the polymer volumetric fraction, which correlates to the amount of non-solvent absorbed by the polymer through Equation (9).

(9) $$\varphi_{3}=-\frac{n_{3}\times V_{3}}{n_{1}\times V_{1}+n_{3}\times V_{3}}$$

where *n*_3_ is the amount of polymer (mol), *V*_3_ is the polymer molar volume (cm^3^∙mol), *n*_1_ is the amount of water (mol), and *V*_1_ is the water molar volume (cm^3^∙mol).

#### 2.6.3. Determination of the Interaction Solvent/Polymer Parameter (*χ*_23_)

**Figure 2:**
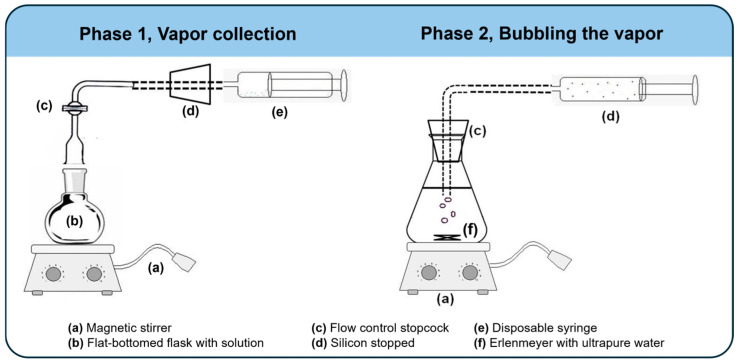
Experimental apparatus for vapor pressure measurements of PA66 solutions and pure solvents. Phase 1 represents the vapor collection, while Phase 2 represents the vapor bubbling in ultrapure water.

The determination of the solvent/polymer interaction parameter (*χ*_23_) was performed through vapor pressure measurements for the solutions and pure solvents in equilibrium [13]. PA66 solutions were prepared by dissolving 5, 10, 15, 20, 25 and 30 g of PA66 in 100 cm^3^ of HCℓ and FA. The vapor was collected from the PA66 solutions and pure solvents in an equipment setup (as shown in Figure 2, Phase 1) for 2 h under magnetic stirring at room temperature (25 ± 2 °C) and 1 atm.

The vapor collected (Figure 2, Phase 1) was bubbled in ultrapure water under magnetic stirring for 10 min, as shown in Figure 2, Phase 2. In sequence, the resulting solution was titrated with 0.09746 mol∙L^−1^ NaOH aqueous solution for bubbled solutions from PA66/HCℓ or 0.01 mol∙L^−1^ NaOH for bubbled solutions from PA66/FA [50].

The stoichiometric ratios were established by quantifying the solvent molar concentration (*n*_1_) in the vapor phase. The vapor pressure of the solutions (*P*_1_) and pure solvents ($P_{1}^{0}$) were determined from the ideal gas equation (*PV = nRT*). With the values obtained, the solvent activity (*a*_1_, Equation (10)) [51], and then, the solvent/polymer interaction parameters (*χ*_23_) were calculated for all concentrations by Equation (11).

(10) $$a_{1}=\frac{P_{1}}{P_{1}^{0}}$$

(11) $$\chi =\frac{\left[\ln\left(\frac{P_{1}}{P_{1}^{0}}\right)-ln\varphi_{1}-\left(1-\frac{1}{N_{2}}\right)\varphi_{2}\right]}{\varphi_{2}^{2}}$$

where *N*_2_ = *V*_2_*/V*_1_, which is the proportion between the molar volumes of the components.

Finally, the data were processed by DataFit software version 7.1.44, and the values for *χ*_23_ given were determined by the non-linear fit coefficient.

## 3. Results and Discussion

### 3.1. PA66 Water Absorption

PA66 absorbed 1.92 ± 0.02% (*w*/*w*) of water, which is explained by the hydrophilic nature of polyamides, absorbing water from the environment through hydrogen bonds [52]. According to Kohan (1995), pure PA66 can absorb up to 8.5% of its weight when immersed in water and up to 2.5% in an environment with 50% relative humidity [2]. Le Huy & Rault (1994) also found a value of 2.5% but under 100% relative humidity [52]. Brandrup & Immergut (1989) observed 1.2% water absorption by PA66 from the brand Zytel (following the ASTM D570 [39]) and concluded that membranes produced with hydrophilic polymers have improved permeability [36].

### 3.2. PA66 Membrane Characterization

Several studies describe preparation of polyamide membranes by immersion precipitation (IP) using formic acid as a solvent [3,23]. However, only a few works on the membranes prepared by the IP method using HCℓ as a solvent are found in the literature [53,54]. In this sense, a complete membrane characterization is relevant to compare the PA66 membranes prepared using HCℓ and FA as solvents.

#### 3.2.1. Chemical Characterization Through Fourier-Transform Infrared Spectroscopy Analysis

**Figure 3:**
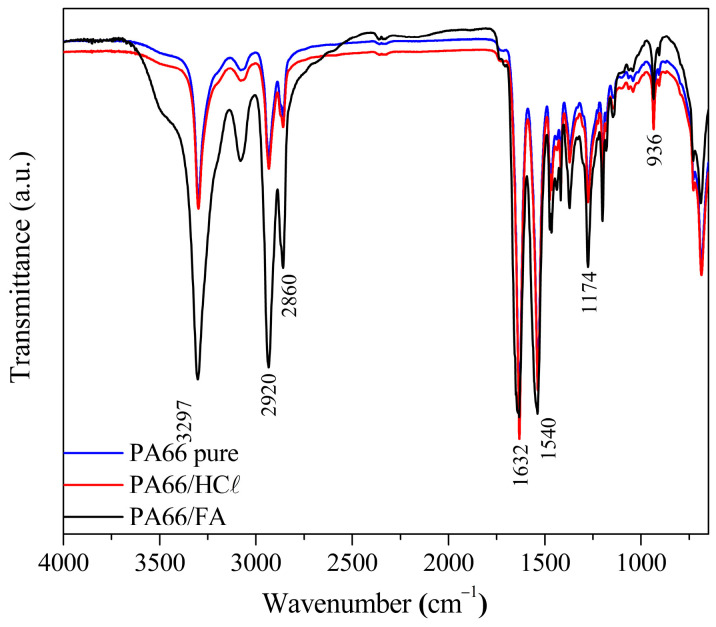
FTIR of PA66/FA and PA66/HCℓ membranes prepared by the immersion precipitation technique and pure PA66 for comparison.

FT-IR analyzes were performed for PA66/HCℓ and PA66/FA membranes, as well as for pure PA66 as a comparison, as seen in Figure 3. Polyamide did not change its structure with the use of solvents, which is evidenced by the presence of PA66 characteristic bands, such as N–H stretching at 3297 cm^−1^, C–H_2_ stretching at 2920 and 2860 cm^−1^, and C=O amide stretching at 1632 cm^−1^. Similar results were described for PA66 membranes prepared using HCℓ as solvent [3,54,55]. The 936 cm^−1^ band is attributed to the PA66 crystalline phase and the trans conformation of the crystalline and amorphous phases, while the bands between 1144 and 1180 cm^−1^ are referred to as N-C=O skeletal vibrations of amorphous phases [56]. The presence of all bands from pure PA66 in PA66/FA and PA66/HCℓ membranes indicates the removal of solvents during the IP method did not modify the polymer structure [41].

#### 3.2.2. Thermal Analysis Through Differential Scanning Calorimetry

**Figure 4:**
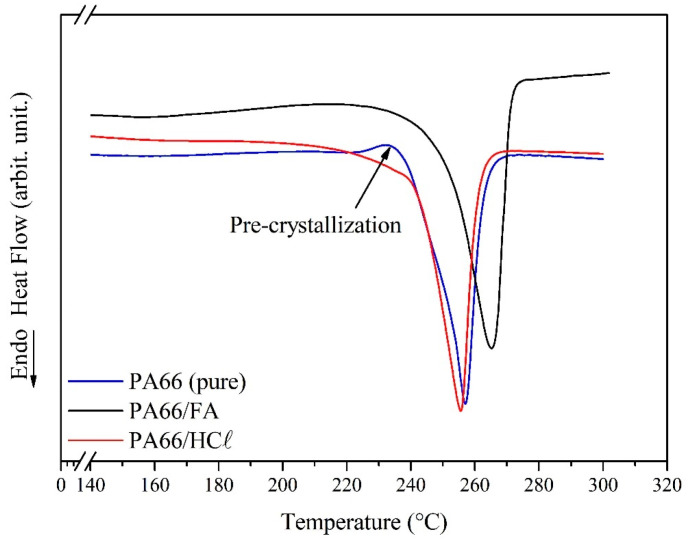
DSC curves for pure PA66, PA66/HCℓ, and PA66/FA membranes.

DSC curves for PA66/HCℓ and PA66/FA membranes for the first heating (and pure PA66, for comparison) are shown in Figure 4, while the results of *T_f_*, Δ*H_f_* and *X_c_* obtained from the curve analysis are presented in Table 3. A single melting event can be observed between 250–265 °C for both samples, which is in agreement with polyamides’ typical melting temperature [57]. The PA66 DSC curve has a discrete pre-crystallization event (~250 °C) [57], which does not appear in the membranes’ curves, probably due to the structural organization caused by the dissolution in the solvents (HCℓ and FA).

No change in the thermal behavior of the PA66/HCℓ membrane was observed when compared to pure PA66, maintaining the same *T_f_* and *X_c_* values. Contrarily, *T_f_* and *X_c_* increased in the PA66/FA membrane (from 256 °C to 265 °C for *T_f_* and from 35 to 45% for *X_c_*). According to Mulder (2003) and Canevarollo (2010), any factor that increases the intermolecular forces will increase the polymer *T_f_*. For the PA66/FA membrane, hydrogen bonds formed between PA66 and FA are responsible for the greater intermolecular forces [6,58].

DSC results proved that the HCℓ acted only as a solvent, not attacking or changing the PA66 structure (observed by the unaltered thermal and crystallinity properties), in the same way as observed by Poletto (2010) [41]. Regarding the effect of these properties on membrane performance, higher crystallinities are correlated to a significant permeability reduction (when compared to amorphous polymeric membranes) [36].

#### 3.2.3. Morphological Analysis of the PA66 Membranes

PA66/FA films became opaque and dropped off the plates within 7–10 s after being immersed in water. PA/HCℓ films started to be turbid during solvent evaporation (60 min in the air), completing the change in appearance when immersed in the precipitation bath (7–10 s). This two-stage phase separation process (a dry–wet phase separation process) is one way to obtain a defect-free top layer (homogeneous) in asymmetric membranes [6].

Visual observation is the most simple technique to distinguish instantaneous (t < 1 s) and delayed L–L demixing processes [6]. The first is characterized by the opaque appearance (instead of translucid) and the instantaneous film displacement from the glass plate when immersed in a non-solvent bath. On the other hand, in delayed L–L demixing, the film takes a certain amount of time to become opaque and come off the glass plate. In this sense, both films can be classified as delayed L–L demixing processes.

**Figure 5:**
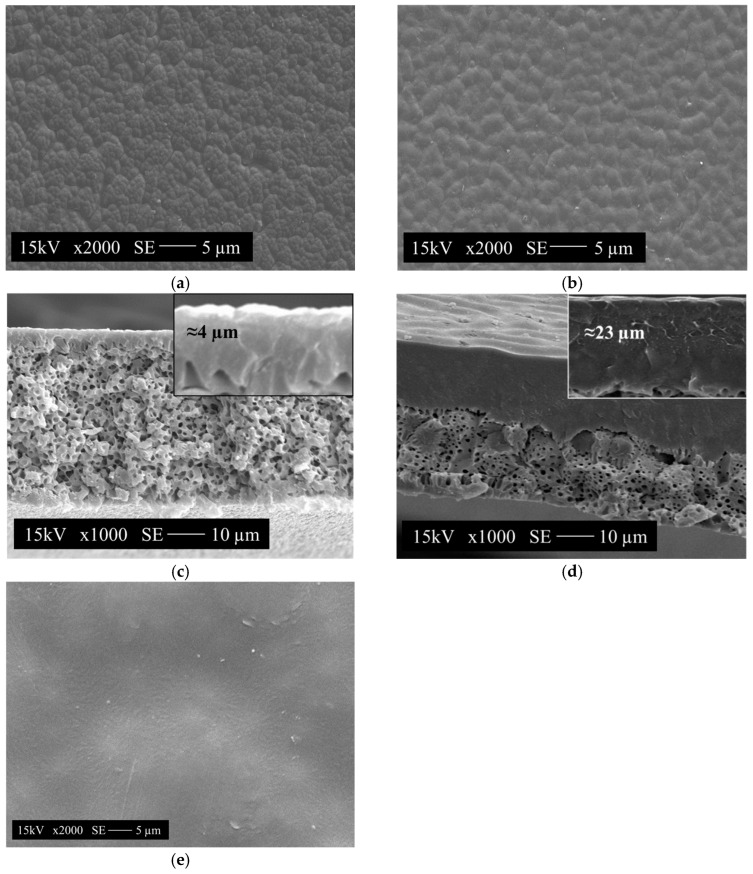
Micrographs of the PA66 membranes. (**a**) PA66/FA and (**b**) PA66/HCℓ upper surfaces (magnification of 2000×). (**c**) PA66/FA and (**d**) PA66/HCℓ transversal sections (magnification of 1000×). (**e**) Detail of the radial spherulites of the PA66/HCℓ membrane’s upper surface (magnification of 2000×).

The SEM images of the PA66 membranes are shown in Figure 5. Both membranes’ (PA66/FA and PA/HCℓ) upper surfaces revealed a non-porous and homogeneous dense layer composed of polygonal grains (Figure 5a,b).

According to the studies of phase demixing mechanisms in PA66/FA membranes [3,25], the polygonal characteristic with quasi-linear limits similar to spherulites is characteristic of the S–L demixing phases process (crystallization). The slow spherulitic crystallization of the polymer crystalline fraction occurs within this layer (skin) in the delayed immersion precipitation phase demixing process. In addition, the crystal grain morphology is limited because of impurities (solvent, non-solvent, and non-crystallizable materials) present in these regions being expelled during the phase of demixing and forming borders.

The (radial) spherulite formation could be observed In the PA66/HCℓ membrane (Figure 5e). The crystalline fraction of aliphatic polyamides favors the spherulite formation because the solution crystallization temperature is lower than the polymer melting temperature [6]. The presence of spherulites (Figure 5a,b) can be correlated to the solutions’ intrinsic viscosity and the viscosimetric radius size of the molecules in the solution. The higher viscosity of the PA66 solutions in FA (*[η]_FA_* = 0.555 dL·g^−1^) with radius expansion of the molecule (*R_V_* = 100.7 nm) provided larger polygonal grains than the PA66 membrane surface in HCℓ (*[η]_HCℓ_* = 0.185 dL·g^−1^ and *R_V_* = 69.4 nm).

The presence of spherulites In the PA66/HCℓ membranes (although smaller than in PA66/FA membranes) may be due to the solvent evaporation step before the precipitation bath due to the higher solvent volatility (277 mmHg) and the additional time allowed for the reorganization of the polymer crystalline fraction. In addition, the spherulites would be larger if HCℓ was pure and not a solution (37%). Poletto (2010) reported an increase in the polygonal grain size due to solvent volatility. Similar results were observed by Guan et al. (2006), which related a better interaction between the solvent and polymer with smaller grain area, uniformity, and less membrane surface irregularity [59].

The cross-sections of the PA66/FA and PA66/HCℓ membranes (Figure 5c,d) have similar morphologies. The morphology of the PA66 membranes in both solvents is typical of PA membranes precipitated in aggressive baths (such as water), with a dense layer and cellular pore structure. The PA66/FA membrane (Figure 5c) has a dense layer of approximately 4 μm and small cellular pores surrounded by the polymeric matrix. On the other hand, the PA66/HCℓ membrane (Figure 5d) has a denser layer (~23 μm) and small cell pores surrounded by the polymeric matrix. Overall, the cross-section images pointed to a phase demixing process initiated by a S–L demixing followed by a L–L demixing process, both delayed and intensely competitive [3,5,25,26].

The formation of a non-porous dense layer (Figure 5) occurs because of the increasing polymer concentration at the interface of the film with the bath (water) [3]. During the precipitation, a thick gel layer was formed on the surface, excluding the possibility of liquid micelles nucleation in the region (pore formation). This process was more intense in the PA66/HCℓ membrane because of the solvent evaporation step starting the precipitation before the immersion bath [6]. This step probably favored the higher polymer concentration on the surface, resulting in a greater dense layer thickness (23 μm) compared to the PA66/FA (4 μm) membrane.

#### 3.2.4. Polymer Binary and Ternary Systems

##### (*g*_12_) the Non-Solvent/Solvent Interaction Parameter

The Gibbs free energy of the mixtures (Δ*G*_m_) obtained by simulation as a function of the volumetric fraction of H_2_O (*φ*_1_) for each system (H_2_O/FA and H_2_O/HCℓ; six diverse experimental points) is presented in Table S1 (Supplementary Information).

**Figure 6:**
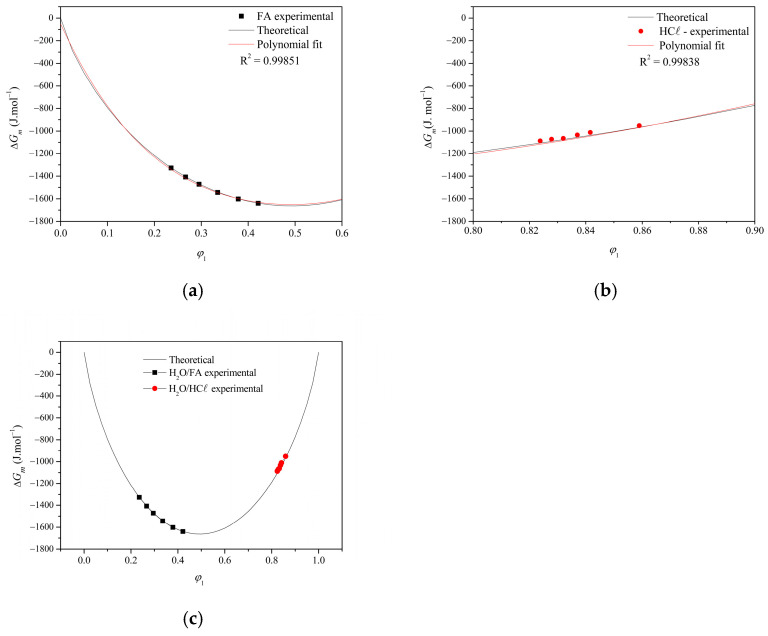
Behavior of the Gibbs free energy of the non-solvent/solvent mixture for (**a**) H_2_O/FA and (**b**) H_2_O/HCℓ (experimental, theoretical and polynomial fit systems). The combined data in (**c**) highlights the position differences between the two systems.

Figure 6 shows the behavior of the Gibbs free energy of the non-solvent/solvent mixture of the experimental, theoretical, and polynomial fit systems for (a) H_2_O/FA and (b) H_2_O/HCℓ. The obtained experimental values agree with the simulated ones with adequate adjustment (based on the *R^2^*). The two systems behave differently: the increase in the volumetric fraction of water promoted a more intense non-solvent/solvent interaction in the H_2_O/FA system (indicated by the higher negative values of Δ*G*_m_), while the opposite was observed in the H_2_O/HCℓ system. This behavior can be better visualized when both data are combined in a single plot, as demonstrated in Figure 6c: the data from the H_2_O/FA system are on the descending part of the parabola and from the H_2_O/HCℓ system on the ascending part. This means that the H_2_O/HCℓ system accepts twice the water fraction of the H_2_O/FA system.

The explanation may be the formation of hydrogen bonds between FA and water in the H_2_O/FA system. Given it is an exothermic process, the H_2_O/FA system is more favorable. On the other hand, the H^+^ ion of the acid from the H_2_O/HCℓ system binds to water molecules and forms a hydronium ion (H_3_O^+^), displacing the equilibrium to the right and reducing the Δ*G_m_* value when compared to the H_2_O/FA system. This process is exothermic and favorable (even to a reduced degree regarding Δ*G_m_* value) but in greater intensity in relation to the water volumetric fraction (*φ*_1_).

**Figure 7:**
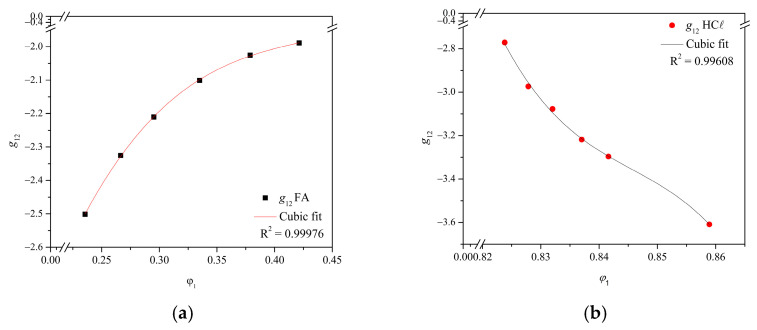
Interaction parameter *g*_12_ (non-solvent/solvent) for (**a**) the H_2_O/FA system and (**b**) the H_2_O/HCℓ system as a function of the H_2_O volumetric fraction (φ_1_).

The *g*_12_ non-solvent/solvent interaction parameters for the H_2_O/FA and H_2_O/HCℓ systems as a function of the H_2_O volumetric fraction (*φ*_1_) are shown in Figure 7. Concentration dependences are provided in Table S2 (Supplementary Information), as calculated by the cubic fit performed for both the H_2_O/FA and H_2_O/HCℓ systems.

The *g*_12_ non-solvent/solvent interaction parameter behaves differently for the H_2_O/FA and H_2_O/HCℓ systems. The *g*_12_ increased with *φ*_1_ for the H_2_O/FA system, while the opposite was observed for the H_2_O/HCℓ, showing the strong interaction between the non-solvent/solvent. The more negative *g*_12_ values for water and HCℓ indicated a more favorable system than the H_2_O/FA [6,44,60]. Negative *g*_12_ values influence the binodal position in the phase inversion process [45].

The cubic fit had an adequate adjustment to the data for both systems, and the differences between them can be associated with the concentrations (37–38% for HCℓ versus 98–100% for FA) and vapor pressure (277 mmHg for HCℓ versus 42 mmHg for FA). Strong interactions between H_2_O and FA were attributed to hydrogen bonding and the partial dissociation of FA in water, which contributes to excess free enthalpy of the mixture and the *g*_12_ interaction parameter [23]. There is no hydrogen bonding between water and HCℓ; however, the acid dissociation constant of HCℓ (*K_a_* = 1 × 10^7^) is higher than FA (*K_a_* = 1.77 × 10^−4^), explaining the *g*_12_ values from the H_2_O/HCℓ system.

Negative values for the *g*_12_ interaction parameter may have influenced the morphology of the prepared films. According to van de Witte et al. (1996), the lower the *g*_12_ value, the longer the film precipitation time required (delayed demixing) and the higher the polymer concentration in the film [5]. Altena & Smolders (1982) related this manifestation to the strong interaction between the non-solvent/solvent requiring a smaller water amount for the L–L phase demixing [45]. Zhang et al. (2011) affirm the solvent amount leaving the film is greater than the non-solvent amount entering the film, resulting in considerably dense layers [47]. This effect was more significant in PA66/HCℓ films (Figure 5c), probably due to the higher vapor pressure of HCℓ.

Nonetheless, considering *g*_12_ as concentration-dependent of the water volumetric fraction added to the ternary system non-solvent/solvent/polymer [6,23,24,45,47], the values obtained for both systems did not differ significantly with the increase of the *φ*_1_ fraction. The polymer concentration range evaluated (10–30 g, moderate concentration) justifies the concentration dependence. It is possible that lower concentrations would intensify the *φ*_1_ concentration dependence in the membrane formation [61], in which more than 10 g of polymer promote a constant ratio between non-solvent and solvent.

##### (*χ*_13_) Non-Solvent/Polymer Interaction Parameter

The *χ*_13_ non-solvent/polymer interaction parameter is constant and concentration-independent [23] and is usually determined by swelling measures in equilibrium [44]. The measurements of H_2_O/PA66 at 25 °C obtained an *χ*_13_ of +2.77. The literature reports *χ*_13_ = +1.4 for the water/cellulose acetate [45], *χ*_13_ = +3.7 for H_2_O/PSU [45], *χ*_13_ = +2.83 for H_2_O/PES [44], and *χ*_13_ = +2.34 for H_2_O/PMMA [60]. Cheng et al. (1994) obtained *χ*_13_ = +1.40 for the H_2_O/PA66 system using a PA66 (Zytel 101, Wilmington, DuPont) with a molar mass of 87,000 g·mol^−1^ and intrinsic viscosity of 2.683 dL·g^−1^ [33] (whereas the PA66 used in this work presents 11,600 g·mol^−1^ molar mass with intrinsic viscosity of 0.555 dL·g^−1^). This result indicates that *χ*_13_ is inversely proportional to the molar mass and the polymer’s intrinsic viscosity.

According to Mulder (2003), a non-solvent penetrates the polymer until the chemical potential of the liquid inside is equal to the chemical potential outside [6]. Thus, the equilibrium establishment may have been impaired by the lower polymer molar mass and enhanced the *χ*_13_ interaction parameter.

##### (*χ*_23_) Solvent/Polymer Interaction Parameter

**Figure 8:**
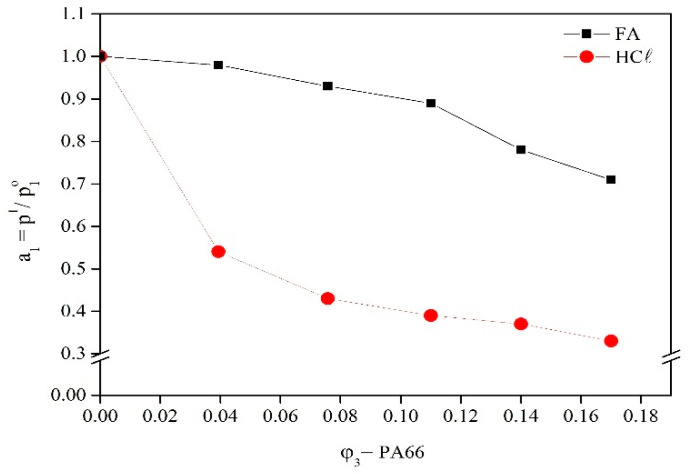
FA and HCℓ activity as a function of the PA66 volumetric fraction (*φ*_3_) in the solution.

The solvent/polymer interaction parameter (*χ*_23_) determination required the study of solvent activity from liquid/vapor equilibrium by means of the vapor pressure method [13,62]. Solvent activity (calculated from Equation (10)) of FA and HCℓ at different PA66 concentrations are presented in Figure 8.

The FA and HCℓ activity as a function of the PA66 volumetric fraction can be explained by the Flory–Huggins theory [63,64]. This theory proposes that polymer solutions have negative deviations from Raoult’s Law and are triggered by wider entropic contributions caused by the difference between the molar volumes of the solvents and polymer. However, the large molar mass difference can be due to the similar vapor pressures of dilute solutions (*P*_1_) and pure solvent ($P_{1}^{0}$) at the same temperature since only the solvents coexist in both phases (given that polymers do not evaporate) [13]. This behavior was observed in the present study for the PA66/FA system but not for the PA66/HCℓ system.

The vapor pressure of pure HCℓ was 277 mmHg, which is the same found value in the literature [65]. The value obtained for FA was 45 mmHg, which is close to the tabulated value in the Handbook of Chemistry and Physics (1962): 42 mmHg [66]. The HCℓ used employed (fuming HCℓ, boiling point = 45 °C [67]) was more volatile than FA (boiling point = 100.7 °C [67]), which was evidenced by the difference in vapor pressure with the addition of PA66 (Figure 8). FA is less volatile than HCℓ (Figure 8), revealing the behavior described by Tager (1978) and Wohlfarth (2001), i.e., a minor difference in the vapor pressure with the polymer addition [12,13].

The solvent activity In the PA66/FA and PA66/HCℓ systems was reduced with the increase of the polymer volumetric fraction, influencing the PA66/HCℓ system more than the PA66/FA system. It can be explained by the strong interaction of HCℓ with PA66 through dipole–dipole intermolecular forces [55], which is similar to the behavior of polyelectrolyte solutions [33]. On the other hand, FA and PA66 interact through hydrogen intermolecular bonds and do not possess the same affinity between solvent and polymer. It can be attributed to the limitation of the Flory–Huggins model in assuming an ideal interaction between polymers and solvent molecules. This model is constrained to liquids without volume variation in the mixture since significant differences in intermolecular forces and molecule size are not considered [32]. Similar behavior was observed by Karimi et al. (2008), which showed the pressure reduction of N, N’-dimethylformamide (DMF, polar solvent) as a function of the polysulfone volumetric fraction in both the semi-dilute and concentrated conditions [14].

The solvent volatility influences the membrane morphology; for instance, volatile solvents form less porous membranes with a higher polymer concentration in the membrane’s upper (dense) layer [59], as observed in Figure 5. FA is less volatile, so the diffusion speed between the solvent and the non-solvent in the immersion–precipitation process is slower, promoting pore formation. HCℓ is more volatile; thus, the diffusion speed in the demixing process is faster, favoring the formation of a non-porous dense layer [3].

**Figure 9:**
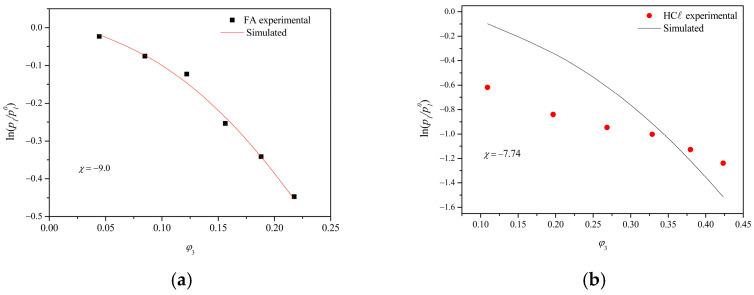
Vapor pressure results and the non-linear fit corresponding to the solvent/polymer interaction parameter (*χ*_23_) for the (**a**) PA66/FA and (**b**) PA66/HCℓ systems (P = 1 atm, T = 25 °C).

The solvent/polymer interaction parameter (*χ*_23_) for the PA66/FA and PA66/HCℓ binary systems was calculated using the vapor pressure results, and the values for *χ*_23_ were given by the non-linear fit coefficient, as shown in Figure 9.

The nonlinear fit using the slope coefficient provided values for the solvent/polymer interaction parameter (*χ*_23_) of −9.0 for the PA66/FA system and −7.74 for the PA66/HCℓ system. The Flory–Huggins interaction parameter (*χ*) is an interaction force measured between solvent and polymer. If *χ* is less than 0.5, there are favorable interactions between solvent and polymer, and the solvent dissolves the polymer [30]. Poor solvents (solvents with a smaller variation of Δ*G* and Δ*μi* when interacting with polymers [68]) have an *χ* value greater than 1.0, while good solvents (solvents with a larger variation of Δ*G* and Δ*μi* when interacting with polymers [68]) have an *χ* with negative values, as observed in the present study. The determination of the solvent activity was made with the interaction parameter *χ*, improving the experimental data interpretations. This is particularly possible for polar systems, given that the Flory–Huggins model considers the entropic term related to the polymeric chain configuration, while the enthalpic term relates to the polymer and solvent molecules interactions [32].

It is possible to observe in Figure 9 that the PA66 volumetric fractions (*φ*_3_) added to the PA66/HCℓ system are superior to the ones added to the PA66/FA system. Moreover, the logarithm values of HCℓ activity are more negative when compared to FA. However, due to the best non-linear fit of PA66/FA system, the value of *χ*_23_ was more negative, indicating that FA is a better solvent for PA66. The inadequate non-linear fit of the PA66/HCℓ system probably occurred due to the acid volatility and the water amount present in the solvent, raising the *χ*_23_ value and suggesting that HCℓ is not as good of a solvent as FA for PA66. Considering the lower HCℓ concentration in the solution (37–38%, compared to 98–100% for FA) and that almost twice the polymer fraction (*φ*_3_) has been dissolved, HCℓ can be considered the best solvent for PA66. Strong acids protonate polyamides and significantly enhance the dissolution of the crystalline phase; however, even though water interacts with polyamides through hydrogen bonding, it cannot be considered a solvent at the evaluated temperature [69].

Cheng et al. (1994) determined *χ*_23_ for Nylon 6, Nylon 66, and Nylon 610 with FA and H_2_O, but using the exothermic interaction of polyamides with FA to build the ternary systems, barring a direct comparison [33]. Later, Bulte et al. (1996) determined *χ*_23_ for the ternary systems of three types of nylon and FA/H_2_O utilizing the Flory–Huggins model [23]. The interaction parameters found were expressed as logarithmic functions of ratios of the volume fractions of solvent–polymer (*v*_2_) obtained from the fusion temperature for Nylon 4,6 (−3.95–1.46 ln*v*_2_), Nylon 4,6-co-6 (−4.32–1.32 ln*v*_2_), and Nylon 6 (−3.91–1.59 ln*v*_2_) [23].

Previous studies demonstrated the Gibbs free energy (Δ*G_m_*) of both homogeneous mixtures is negative [43]. Negative values for *χ*_23_ were obtained for the PA6/poly(vinyl alcohol)(PVA)/FA system in different concentrations, with −1.7846 being the most negative value for the PA6/PVA 20/80 concentration [55]. The negative values of *χ*_23_ indicate that the miscibility is attributed to hydrogen bonds between the PA6 and PVA segments [55]. The PA66/PVA/FA system in different concentrations also had negative *χ*_23_ values, with *χ*_23_ = −14.1 for the PA66/PVA 50/50 concentration [70]. The authors attributed the reduction of the hydrogen interactions between PA and segments of PVA when the ratio of hydrocarbon chain to amide group increases from six carbons in PA66 [70]. This value (*χ*_23_ = −14.1) is more negative than the one obtained in the present work for the PA66/FA and PA66/HCℓ systems. It is believed the negative values for the *χ*_23_ of polyamides are due to the polymer protonation by solvents.

Low values for *χ*_23_, coupled with high values of *χ*_13_, increase the amount of non-solvent necessary for the L–L phase demixing process [45]. The PA66/HCℓ system presented this behavior; aside from the solvent containing a large water concentration in its formulation, the added amount of non-solvent for precipitation was practically double the required amount for the PA66/FA system (in which the solvent is virtually pure).

##### Ternary Systems

**Figure 10:**
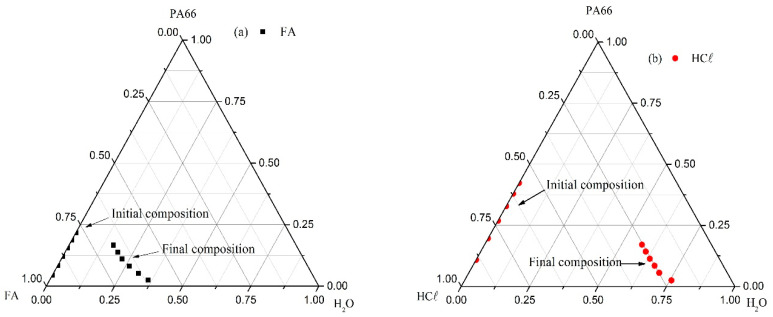
Ternary phase diagrams comparing the initial composition (binary system) with the cloud point curve (*χ*_23_ final compositions) for the (**a**) H_2_O/FA/PA66 and (**b**) H_2_O/HCℓ/PA66 systems.

The ternary phase diagrams comparing the initial composition (binary system) with the cloud point curve (*χ*_23_ final composition) for the H_2_O/FA/PA66 (a) and H_2_O/HCℓ/PA66 (b) systems are shown in Figure 10. Thermodynamic systems are generally demonstrated by a ternary phase diagram, which comprises non-solvent, solvent, and polymer (here, H_2_O, FA or HCℓ, and PA66, respectively). The triangle vertices represent the pure components, and the points inside the triangle represent the mixture of three components. The boundary of the L–L phase demixing is generally called the “binodal curve” for monodisperse polymers, whereas it is called the “cloud point curve” for polydisperse polymers [5].

Analyzing Figure 10a, the cloud point curve for the ternary system H_2_O/FA/PA66 is close to the solvent/polymer axis (FA/PA66), in which the system’s initial composition can be found. It indicates that less non-solvent is required to induce the phase demixing [47], intensifying as the PA66 concentration increased, which is similar to that observed by Zhang et al. (2011) [47]. It is also in agreement with the presence of a thick dense layer (Figure 5c,d) and the intersection point of the phase demixing on the non-solvent/polymer located near the polymer axis since higher *χ*_13_ values are attributed to a delayed phase demixing process [5].

On the other hand, the cloud point curve for the ternary system H_2_O/HCℓ/PA66 (Figure 10b) is close to the non-solvent/polymer axis (H_2_O/PA66) and far from its initial composition, indicating that a larger water volumetric fraction (*φ*_1_, non-solvent) was required to induce the phase demixing. This effect was also confirmed by the *φ*_1_ added to the non-solvent/solvent binary system (*g*_12_, H_2_O/HCℓ), which was practically double the added amount to the H_2_O/FA system.

According to Mulder (2003), the cloud point curve is the boundary between the one-phase region (homogeneous and equilibrium) and the two-phase region [6]. This means that the homogeneous region of the PA66/HCℓ binary system is larger than the PA66/FA binary system, favoring a membrane formation with uniform microstructure [47].

Lower *g*_12_ values indicate high miscibility between non-solvent and solvent, reducing the solvent strength because the non-solvent competes with the polymer for the solvent [60]. This effect was not observed in the H_2_O/HCℓ/PA66 ternary system. Although it has presented a lower *g*_12_ value, the solvent strength was not reduced (cloud point curve location in Figure 10b), which may be justified by the HCℓ ionization force (*K_a_* = 1 × 10^7^).

**Figure 11:**
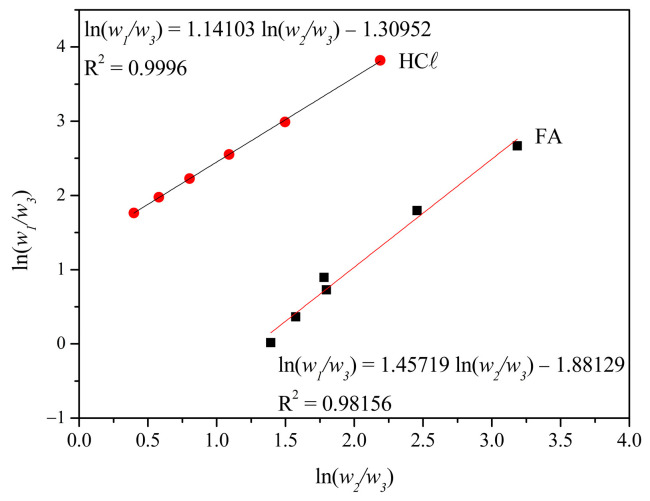
Cloud point curve linearization for the H_2_O/FA/PA66 and H_2_O/HCℓ/PA66 systems based on CPL Boom’s correlation.

The cloud point curve linearization using Boom’s CPL method (Equation (6)) for the H_2_O/FA/PA66 and H_2_O/HCℓ/PA66 systems is shown in Figure 11.

The H_2_O/FA/PA66 system presented perfectly adjusted linearization (*R*^2^ = 0.9996), indicating that L–L demixing occurred (according to the parameter of *R*^2^ > 0.999 [47]). PA6 and PA66 solutions in strong acids (such as H_2_SO_4_ and HCℓ) have an L–L phase demixing structure [33], similar to the one observed in Figure 5d when immersed in water. This is due to the strong protonation of the polyamide, which remains even in the presence of water. As a result, the crystal nucleation and growth are delayed and the L–L phase demixing becomes kinetically favored in relation to the S–L crystallization process.

The H_2_O/FA/PA66 system shows a lower *R*^2^ fit deviation slightly below 0.999 (0.98156), indicating that both L–L and S–L (or crystallization) demixing processes occurred. While some studies stated that the PA66/FA/H_2_O system is dominated by the S–L crystallization process [33], others reported competition between L–L and S–L demixing processes [11]. In the latter, the L–L was dominant at lower concentrations (<17% *v*/*w*), while S–L demixing was preferable between 20 and 30% (*v*/*w*) due to the smaller non-solvent amount required for the demixing process, confirming the result found in this work [11].

**Figure 12:**
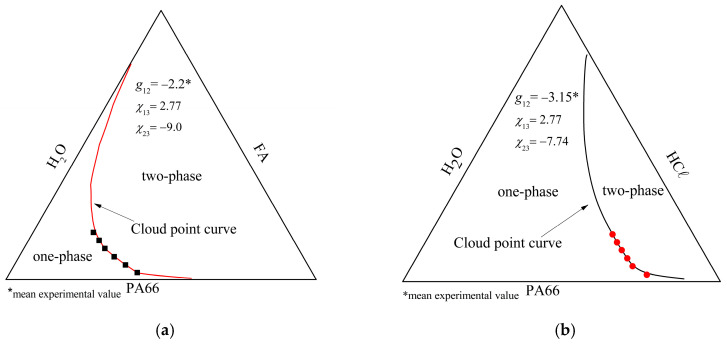
Cloud point curve experimental points and the theoretical curve calculated using the CPL Boom’s correlation for the (**a**) PA66/FA/H_2_O and (**b**) PA66/HCℓ/H_2_O systems.

The cloud point curve experimental points and the theoretical curve calculated using the CPL Boom’s correlation for the PA66/FA/H_2_O and PA66/HCℓ/H_2_O systems are presented in Figure 12. It is observed that the equilibrium region is larger in the PA66/HCℓ/H_2_O system (Figure 12b), indicating that the system accepts more water, probably due to the interactions that occur in the system. The non-solvent competes with the polymer for the solvent, as indicated by the *g*_12_ negative value [60]. No hydrogen bonds happened with PA66/HCℓ/H_2_O, neither between non-solvent/solvent nor between solvent/polymer; but, dipole–dipole interactions, acid–base interactions, and/or similar behavior to polyelectrolyte solutions may have happened [33]. It is likely that the competition for the solvent was not intense because the solvent in this case is a solution (37–38%), i.e., it has a certain amount of water.

For the PA66/FA/H_2_O system (Figure 12a), the stable region is close to the polymer/solvent axis, indicating that a small non-solvent amount is sufficient to promote phase demixing [27,71]. Hydrogen bonds occur both between the non-solvent/solvent and between solvent/polymer, justifying competition for the solvent between the non-solvent and the polymer (*g*_12_ mean value of −2.2) [60].

**Figure 13:**
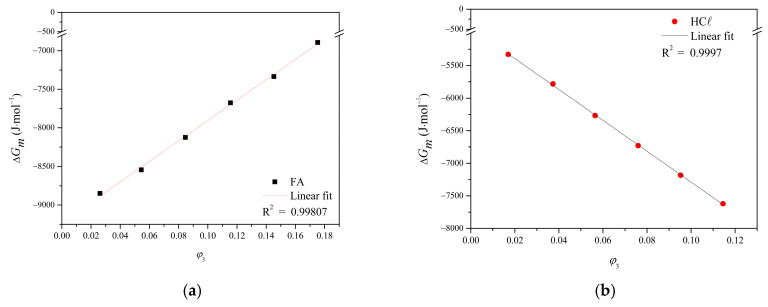
Gibbs free energy of the mixture for the (**a**) PA66/FA/H_2_O and (**b**) PA66/HCℓ/H_2_O ternary systems as a function of the polymer volumetric fraction.

The Gibbs free energy calculated by the Flory–Huggins method (Equation (7)) is shown in Figure 13 as a behavior of the mixture as a function of the polymer volumetric fraction (*φ*_3_) for the PA66/FA/H_2_O and PA66/HCℓ/H_2_O ternary systems. The PA66/FA/H_2_O and PA66/HCℓ/H_2_O ternary systems present opposite behaviors. While Δ*G_m_* increases with *φ*_3_ for the PA66/FA/H_2_O system, it decreases for the PA66/HCℓ/H_2_O ternary system, probably due to interaction forces. Hydrogen bonds among the three components of the PA66/FA/H_2_O system cause a reduction in the spontaneity as *φ*_3_ increases, which is common since polymer and non-solvent compete for hydrogen bonds with the solvent. In the PA66/HCℓ/H_2_O system, dipole–dipole interactions happen between solvent and non-solvent, hydrogen bonds between non-solvent and polymer, and between solvent and polymer the possibilities are dipole–dipole, acid–base, and/or similar behavior to a polyelectrolyte solution [33]. This means the chemical affinity among the three components increases the possibilities of interactions, favoring the system’s spontaneity with an increase in *φ*_3_.

The Flory–Huggins model does not consider all the complexities of specific interactions between solvent and polymer (such as hydrogen bonds and specific intermolecular bonds) [32], which can explain the behavior observed in Figure 12 and Figure 13. However, this shortcoming can be addressed by employing empirical parameters, which do not compromise the simplicity, broad acceptance, and ease of application of the Flory–Huggins model.

## 4. Conclusions

This work investigated two ternary systems (PA66/FA/H_2_O and PA66/HCℓ/H_2_O) using experimental analysis associated with theoretical data regarding the preparation of polymeric membranes using the phase inversion technique. Both the H_2_O/FA/PA66 and H_2_O/HCℓ/PA66 ternary systems promoted similar morphologies to the membranes, which included a dense and non-porous upper layer. The membranes obtained from the H_2_O/FA/PA66 ternary system presented a delayed phase demixing, with L–L and S–L demixing processes competing depending on the polymer concentration. The H_2_O/HCℓ/PA66 membranes also showed delayed phase demixing, following a L–L demixing process.

Regarding the thermodynamic analysis, the Flory–Huggins theory served as a guide; however, several adjustments had to be considered due to the strong polarity of both solvents since the theory was developed for apolar systems. Although both systems were favorable, the Gibbs free energy of the H_2_O/FA/PA66 ternary mixture indicated that this system was more favorable than the H_2_O/HCℓ/PA66 system. In addition, the solvent/polymer interaction parameter of the PA66/FA system indicated that FA is a better solvent. However, considering the HCℓ concentration (37–38%), water amount, volatility, and the dissolved *φ*_3_ fraction, HCℓ can be considered the best solvent for PA66 to produce porous polymeric membranes with a dense upper layer.

Further studies are still necessary to define the metastable region of the ternary system, including the spinodal curve and tie lines, as well as to identify the critical point. This information will enable the quantification of the polymer-rich and polymer-lean phases, which are valuable pieces of information for membrane preparation. Additionally, it is important to develop a methodology to experimentally determine the non-solvent/solvent interaction parameter for both systems.

## Figures and Tables

**Table 1:** Properties of the polymer and solvents employed in this work.

Materials	Molar Volume (cm^3^∙mol^−1^)	Molar Mass (g∙mol^−1^)	Density (g∙cm^−3^)
PA 66	10,642.20	11,600 ^a^	1.22 ^b^/1.09 ^c^
HCℓ (37–38%)	30.67	36.5	1.19
FA (98–100%)	37.7	46	1.22

^a^ viscosimetric average molar mass (*M_v_*) [38]. ^b^ PA66 solid density (arranged) [36]. ^c^ PA66 density in solution (amorphous) [36].

**Table 2:** Conditions of time and temperature for the evaporation and immersion precipitation baths for the preparation of PA66 membranes.

Membrane Solvent	Evaporation		Precipitation Bath	
	Time (min)	Temperature (°C)	Time (min)	Temperature (°C)
FA	10	20	120	25 ± 2
HCℓ	60	60	120	25 ± 2

**Table 3:** Thermal characteristics and crystallinity degree of pure PA66 and PA66/HCℓ and PA66/FA membranes.

Polymer/Membranes	*T_f_* (°C)	Δ*H_f_* (J·g^−1^)	*X_c_* (%)
PA66	256.80	68.86	35
PA66/FA	265.20	88.78	45
PA66/HCℓ	255.51	69.72	35

## Data Availability

The data are contained within the article or Supplementary Materials.

## Supplementary Materials

The following supporting information can be downloaded at: https://www.mdpi.com/article/10.3390/membranes15010007/s1, Figure S1: Schematic of the membrane preparation by the immersion precipitation technique; Table S1: Values of Δ*G_m_* as a function of *φ*_1_ for the H_2_O/FA and H_2_O/HCℓ systems; Table S2: Concentration dependence summary of the (*g*_12_) non-solvent/solvent interaction parameter for the H_2_O/FA and H_2_O/HCℓ systems.
