# Supplementary material for: Investigation of the Ternary System, Water/Hydrochloric Acid/Polyamide 66, for the Production of Polymeric Membranes by Phase Inversion
Source: Membranes (Basel). 2025 Jan 1;15(1):7. doi: 10.3390/membranes15010007 (PMC11767114; doi:10.3390/membranes15010007)
Supplement: Supplementary file 1 [file membranes-15-00007-s001.zip › membranes-3355244-supplementary.pdf]

Supplementary Information

Investigation of the ternary system water/hydrochloric acid/polyamide 66 for the production of polymeric membranes by phase inversion

Jocelei Duarte[1]*, Camila Suliani Raota[2], Camila Baldasso[1], Venina dos Santos[1], and Mara Zeni[1]

[1] Postgraduate Program in Process and Technologies Engineering (PGEPROTEC), University of Caxias do Sul, Caxias do Sul, RS, Brazil
[2] Postgraduate Program in Materials Science and Engineering (PPGMAT),
University of Caxias do Sul, Caxias do Sul, RS, Brazil

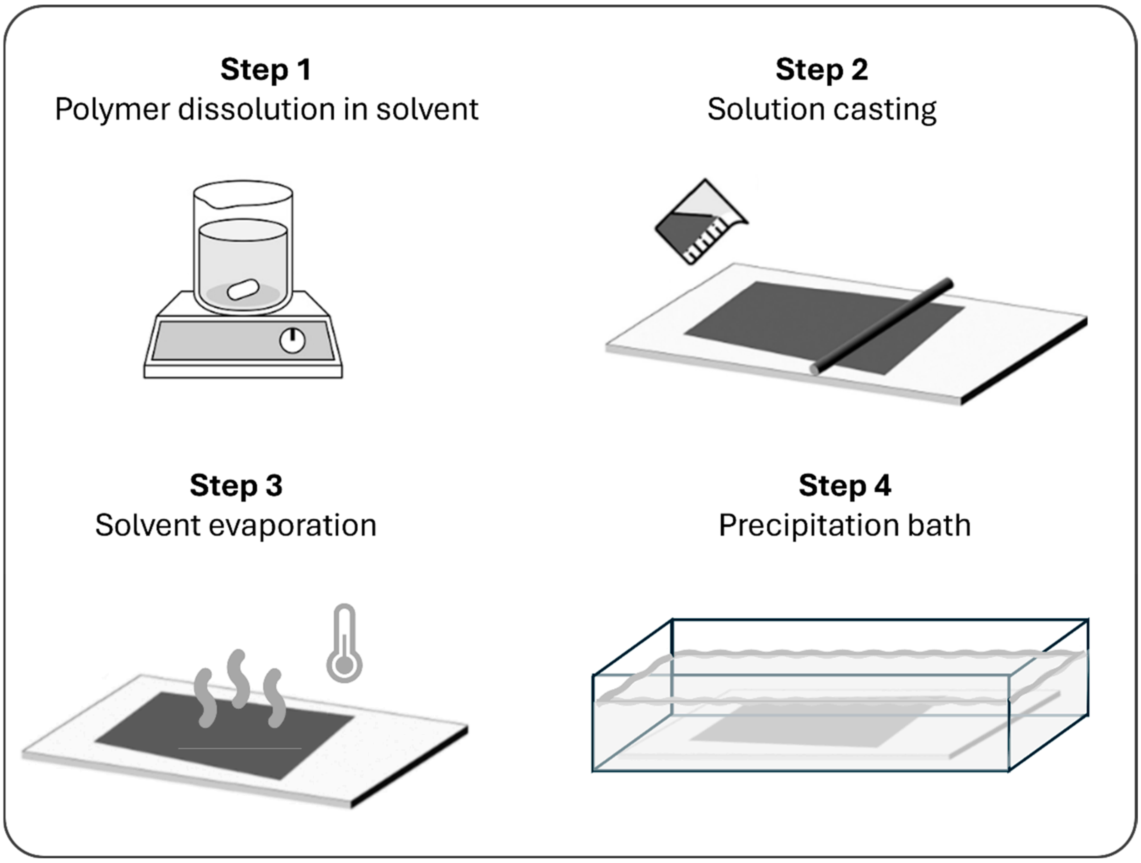

Figure S1. Schematic of the membrane preparation by immersion precipitation technique.

Table S1. Values of ΔG_m as a function of $_1$ for the H_2O/FA and H_2O/HC systems.

H_2O/FA		H_2O/HC	
φ_1 (H_2O)	ΔG_m (J·mol^{-1})	φ_1 (H_2O)	ΔG_m (J·mol^{-1})
0.235637	−1327.35	0.82381	−1086.66
0.266501	−1407.35	0.827854	−1071.62
0.29522	−1472.11	0.832018	−1064.1
0.334987	−1544.49	0.83702	−1034.02
0.378801	−1601.63	0.841598	−1011.46
0.421286	−1639.72	0.858912	−951.28

Table S2. Concentration dependence summary of the (g_{12}) non-solvent/solvent interaction parameter for the H_2O/FA and H_2O/HC systems.

			**FA**			
**Cubic fit**	$-6.24767 + 28.08873\varphi_1 - 63.18037\varphi_1^2 + 48.67504\varphi_1^3$					
**H_2O fraction (φ_1)**	0.235637	0.266501	0.29522	0.334987	0.378801	0.42128
g_{12}	−2.50	−2.33	−2.21	−2.10	−2.03	−1.99
			**HC**			
**Cubic fit**	$15261.5383 - 54129.187\varphi_1 + 63997.7629\varphi_1^2 - 25228.632\varphi_1^3$					
**H_2O fraction (φ_1)**	0.82381	0.82785	0.83202	0.83702	0.8416	0.85891
g_{12}	−2.77	−2.97	−3.08	−3.22	−3.30	−3.60
